# Serum Uric Acid Level Is Positively Associated With Higher Bone Mineral Density at Multiple Skeletal Sites Among Healthy Qataris

**DOI:** 10.3389/fendo.2021.653685

**Published:** 2021-03-31

**Authors:** Wisam Nabeel Ibrahim, Nadin Younes, Zumin Shi, Marawan Abdelhamid Abu-Madi

**Affiliations:** ^1^ Department of Biomedical Sciences, College of Health Sciences, QU Health, Qatar University, Doha, Qatar; ^2^ Biomedical and Pharmaceutical Research Unit, QU Health, Qatar University, Doha, Qatar; ^3^ Biomedical Research Center, Qatar University, Doha, Qatar; ^4^ Department of Human Nutrition, College of Health Sciences, QU Health, Qatar University, Doha, Qatar

**Keywords:** uric acid, bone mineral density, osteoporosis, Qatar biobank, antioxidant, eGFR, BMI, smoking

## Abstract

**Background:**

Oxidative stress has been implicated as a fundamental mechanism in the decline of bone mass. Although serum uric acid (SUA) has potent antioxidant properties, the findings of many epidemiological and experimental studies couldn’t draw a clear conclusion on the relation between SUA and bone health. We aim to investigate the association between SUA and bone mineral density (BMD) at different skeletal sites among healthy Qataris.

**Methodology:**

A cross-sectional analysis including total-body and site-specific bone mineral density scores and other serological markers of 2981 healthy Qatari adults (36.4 ± 11.1 years) from the Qatar biobank database was conducted. The study participants were divided into quartiles based on the level of SUA, and the BMD was measured using dual-energy X-ray absorptiometry (DXA). Multiple regression analyses were applied to investigate the association between SUA and BMD adjusting for multiple confounding factors.

**Results:**

High levels of SUA were significantly associated with the increased bone mineral density of the total body and at site-specific skeletal locations after adjusting for age and gender (p-value < 0.001). Further adjustment for body mass index (BMI), smoking, vitamin D, alkaline phosphatase, and estimated glomerular filtration rate (eGFR) levels attenuated the association but the association remained significant for individuals with high SUA levels (p-value ≤ 0.01).The association between SUA and BMD was not significant in non-obese, females, young adults, and smokers. However, no interaction was found between SUA and age, gender, BMI and smoking.

**Conclusion:**

Higher SUA levels are associated with a high bone density among healthy Qatari adults. However, such observation demands further investigations to outline the underlying mechanisms.

## Introduction

Disorders of bone mineral density are common risk factors that are ranging from moderate osteopenia to severe osteoporosis (1). Osteoporosis is a chronic systemic disorder in the skeletal system characterized by a pathological reduction in bone density (less than 2.5 standard deviations from normal adults) with compromised bone strength [National Institute of Health, 2001]. Each year, 200 million individuals worldwide are diagnosed with osteoporosis with complicated 9 million pathological fracture cases (2). Fractures are the most common cause of physical disability and may confer substantial risk for morbidity and mortality among patients with osteoporosis; commonly involving the hip, wrist, or spine bones (3).

The risk factors for having low BMD include smoking, malnutrition, low calcium intake, steroid medications, physical inactivity, alcoholism, and underweight (4). Other non-modifiable risk factors include old age, female gender, and family history of osteoporosis (5).

Generally, the etiology of osteoporosis involves imbalances between the bone-building osteoblasts and the bone-resorbing osteoclasts leading to significant loss of bone matrix components (4). A large body of scientific evidence has confirmed the role of oxidative stress in the age-related decline of bone mass and strength (6). A high level of reactive oxygen species or low level of antioxidants is linked with reduced bone density and osteoporosis (6–9). These antioxidants had demonstrated osteoprotective properties mainly through maintaining the survival and activity of osteoblasts with inhibition of osteoclast cell activity (10).

Serum uric acid (SUA) is the final product of purine nucleosides and free bases degradation in humans and higher primates with potent antioxidant properties (11). However, when present at abnormally high levels it may become a risk factor for many metabolic syndromes and diseases (12–15). SUA is a powerful scavenger of free radicals, which may contribute as an endogenous systemic antioxidant in protecting the bones from deterioration (16). Many experimental and epidemiological studies provided conflicting conclusions about the relation between SUA and bone health (17, 18). Thus, it is hypothesized that SUA is associated with surrogate markers of bone health, leading to speculation about a potential protective role of SUA against bone loss and metabolic bone diseases, such as osteoporosis, owing to its potent antioxidant effects. Therefore, this study was undertaken to retrospectively investigate the correlation of SUA with BMD in a healthy Qatari population.

## Methodology

### Ethical Statement

The study was ethically approved by the institutional review board (E -2018-QBB-RES-ACC-0112-0054). All samples used in this study were obtained from Qatar Biobank. Written informed consent has been taken from all participants enrolled in the study.

### Subjects

2,981 healthy Qataris aging from 18 to 70 years old volunteered in this study. Samples collection were conducted in Qatar Biobank. A consent from was obtained from all volunteers to use their samples and health information in complete confidentiality by the approved researchers. In addition, all participants signed consent forms agreeing to allow the tracking and assessment of their medical history. The questionnaire information was collected in the interview by a nurse to ensure the accuracy of the patient medical history.

Participants with conditions that might affect bone mass, structure, or metabolism were excluded to minimize their influence on bone mineral density. Patients on corticosteroids treatment for any cause and patients receiving chemotherapy for cancer; chronic disorders involving the vital organs (heart, lung, liver, kidney, brain), history of diabetes, high cholesterol level, high blood pressure, kidney diseases, stroke, arthritis, osteoporosis, fractures, Parkinson disease, thyroid disease, hysterectomy, Hodgkin lymphoma, breast, prostate, and lung cancers were all excluded. The background questionnaire was self-administered and designed to obtain personal socio-demographic data including age, height, weight, and body mass index (BMI). Data regarding the co-morbidities/disease history of the participants and medication or supplement intake were also included in the questionnaire.

### Biochemical Investigations

A routine blood biochemistry profile was performed, which included serum uric acid (SUA), calcium, phosphorus, alkaline phosphatase (ALP), serum creatinine, thyroid hormones test, oestrogen, and serum 25-hydroxyvitamin D. The estimated glomerular filtration rate (eGFR) was determined according to the serum creatinine level, and gender following published method of calculation (19).

### Dual-Energy X-Ray Absorptiometry (DXA) Scan

The BMD data for all study participants were obtained from Qatar Biobank using Full-body dual-energy iDXA (General Electric, Boston, MA, USA). A trained and certified technician in QBB measured the BMD. The total body BMD in addition to multiple site-specific scans including the lumbar spine (L1-L4), pelvis, trunk, femoral neck, trochanteric, and ward’s triangle were all measured. The DXA scan results were reported as absolute values of BMD (g/cm^2^). The same DXA machine was used for the screening of all participants and all technicians received training to ensure the reproducibility of the BMD measures.

### Statistical Analysis

The statistical analysis was done using Stata 16 (Stata Statistical Software: Release 16. College Station, TX: Stata Corp LLC, USA). Data were expressed as mean values ± SD or frequencies (%). Univariate analysis were completed using One way ANOVA and Chi-square tests as appropriate. The unadjusted relation between total body BMD and SUA level was assessed by Pearson correlation analysis. Multiple regression model analysis was conducted to evaluate the strength of association between SUA and site-specific BMD after adjustments with different confounding factors. Statistical significance was indicated with p-values less than 0.05.

## Results

### The Baseline Characteristics According to Serum Uric Acid Quartile Level

The distribution of demographic, biochemical, and bone mass characteristics according to the SUA quartile levels is shown in **Table 1**. A total of 2,981 Qatari participants were included in the study. The mean age was 36.4 ± 11.1 years. The mean SUA level was 296.53 ± 81.89.

**Table 1:** Sample characteristics gender-specific quartiles of uric acids among participants attending Qatar Biobank Study (N = 2981).

	Total	Q1	Q2	Q3	Q4	p-value
	N = 2,981	N = 759	N = 744	N = 743	N = 735	
Age (years)	36.4 (11.1)	34.9 (10.7)	35.7 (10.7)	36.3 (11.0)	38.6 (11.6)	<0.001
Sex (%)						1.00
Men	1,430 (48.0%)	362 (47.7%)	359 (48.3%)	355 (47.8%)	354 (48.2%)	
Women	1,551 (52.0%)	397 (52.3%)	385 (51.7%)	388 (52.2%)	381 (51.8%)	
Smoking (%)						0.38
Non-smoker	2,022 (67.8%)	491 (64.7%)	507 (68.1%)	511 (68.8%)	513 (69.8%)	
Ex-smoker	189 (6.3%)	54 (7.1%)	52 (7.0%)	42 (5.7%)	41 (5.6%)	
Current smoker	770 (25.8%)	214 (28.2%)	185 (24.9%)	190 (25.6%)	181 (24.6%)	
Uric acid (µmol/L)	296.53 (81.89)	222.59 (52.09)	272.40 (50.16)	312.00 (54.87)	381.69 (71.30)	<0.001
BMI (kg/m^2^)	28.4 (5.9)	25.9 (5.2)	27.2 (5.2)	29.1 (5.4)	31.6 (6.0)	<0.001
Alkaline phosphatase (U/L)	68.76 (19.91)	65.69 (21.45)	66.42 (17.01)	71.35 (20.92)	71.68 (19.21)	<0.001
Calcium (mmol/L)	2.28 (0.08)	2.27 (0.08)	2.28 (0.07)	2.28 (0.08)	2.29 (0.08)	<0.001
Phosphorus (mmol/L)	1.15 (0.17)	1.16 (0.17)	1.15 (0.16)	1.15 (0.16)	1.15 (0.17)	0.72
Dihydroxyvitamin D (ng/mL)	18.67 (11.57)	18.33 (11.69)	18.31 (10.23)	18.83 (11.52)	19.22 (12.73)	0.38
eGFR (mL/min/1.73m^2^)	111.35 (15.07)	115.20 (13.79)	112.45 (14.65)	111.19 (14.01)	106.42 (16.41)	<0.001
eGFR< 90 mL/min/1.73m^2^ (%)	256 (8.6%)	40 (5.3%)	50 (6.7%)	54 (7.3%)	112 (15.3%)	<0.001
Estradiol (pmol/L)	237.76 (283.47)	275.70 (326.71)	252.26 (300.27)	225.26 (261.94)	195.93 (226.65)	<0.001
Free Thyroxin (pmol/L)	13.18 (1.59)	13.17 (1.52)	13.28 (1.71)	13.12 (1.57)	13.16 (1.55)	0.25
Free triiodothyronine (pmol/L)	4.43 (0.61)	4.37 (0.57)	4.46 (0.66)	4.44 (0.64)	4.45 (0.56)	0.022
Thyroid Stimulating Hormone (mIU/L)	1.61 (1.15)	1.53 (0.92)	1.60 (1.18)	1.70 (1.38)	1.62 (1.05)	0.047
*Bone mineral density (BMD) score (g/cm^2^)*						
Total body	1.21 (0.13)	1.18 (0.13)	1.20 (0.13)	1.21 (0.12)	1.24 (0.15)	<0.001
Hip trochanter	0.82 (0.14)	0.79 (0.14)	0.81 (0.14)	0.83 (0.13)	0.86 (0.14)	<0.001
Trunk	1.00 (0.12)	0.98 (0.12)	1.00 (0.12)	1.01 (0.11)	1.03 (0.12)	<0.001
Femoral upper neck	0.85 (0.16)	0.83 (0.16)	0.84 (0.16)	0.85 (0.16)	0.87 (0.17)	<0.001
Ward’s triangle	0.83 (0.16)	0.81 (0.16)	0.83 (0.16)	0.84 (0.16)	0.86 (0.17)	<0.001
Pelvis	1.01 (0.13)	0.99 (0.14)	1.00 (0.13)	1.01 (0.12)	1.04 (0.14)	<0.001
Lumber spine	1.11 (0.14)	1.08 (0.15)	1.11 (0.14)	1.12 (0.13)	1.15 (0.14)	<0.001

Data are presented as mean (SD) for continuous measures, and n (%) for categorical measures.

The total body and site-specific BMD including the hip trochanter, trunk, femoral upper neck, ward’s triangle, pelvis, and Lumber spine demonstrated dose dependant response across the quartiles of SUA in the unadjusted analysis. Further, there was a strong association between participants age, BMI, alkaline phosphatase, calcium, oestradiol, eGFR, and the quartile levels of SUA (p<0.01). Each of these variables were positively related with quartiles of SUA level; except for the oestradiol level that had an inverse association with SUA quartiles. A weak association was also evident between SUA quartiles and triiodothyronine level. Dihydroxyvitamin D, smoking, free thyroxin, and phosphorous levels were not associated at all with the quartile levels of SUA as shown in **Table 1**.

### Association Between Serum Uric Acid and Bone Mineral Density

**Figure 1:**
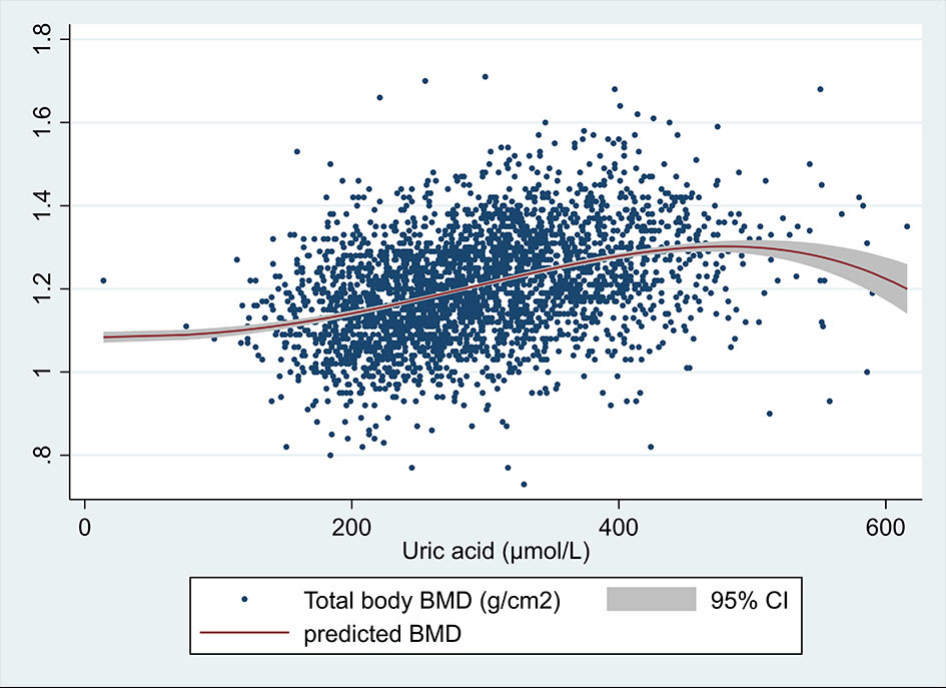
Scatter plot of serum uric acid (µmol/L) and total body BMD (g/cm^2^). The unadjusted prediction was made using the fractional polynomial method. One outlier was removed (total BMD >3).

**Figure 2:**
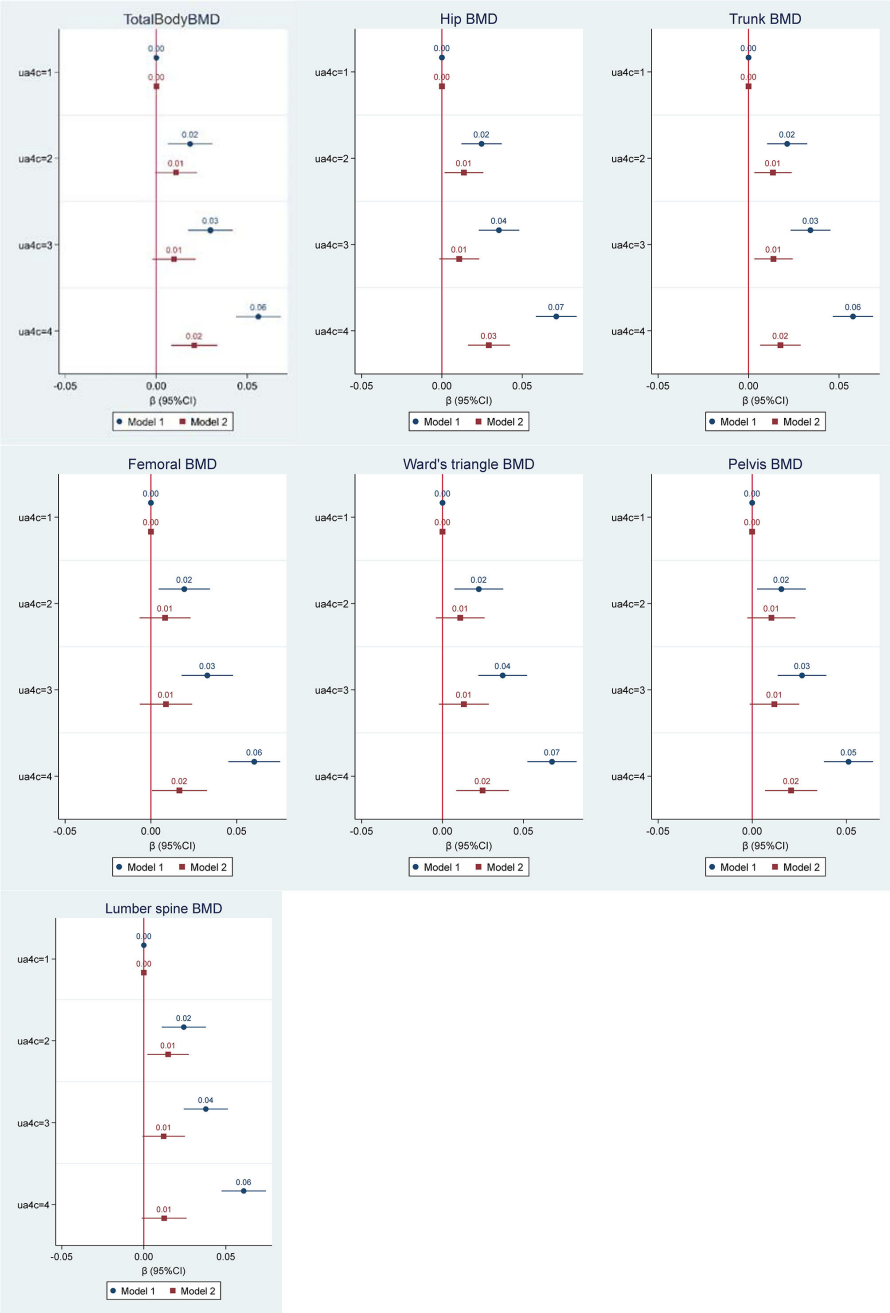
Multiple regression analysis of the association between quartiles of serum uric acid (µmol/L) and bone mineral density (BMD) for total body and multiple skeletal locations (g/cm^2^). Values represent the regression coefficients per SD change of uric acid. Model 1 adjusted for age and gender. Model 2 adjusted for age, gender, BMI, serum vitamin D, Alkaline Phosphatase, eGFR, and smoking status.

A significant positive correlation was found between SUA and total body BMD as shown in the scatter plot in **Figure 1**. **Figure 2** shows the multiple variable adjusted associations between quartiles of SUA and total body and site-specific BMDs.

The first model demonstrated a significant dose-response relationship between serum uric acid and all BMD scores (total body and all site-specific) after adjustment for age and gender. Across quartiles of SUA from low to high, total body and all site-specific BMDs increased. In the second model of adjustments for confounders including age, gender, BMI, serum vitamin D, Alkaline Phosphatase, estimate glomerular filtration rate (eGFR), and smoking status, the above associations were attenuated. However, compared with the first quartile of SUA, the highest quartile had a significantly higher BMD including total body BMD and all site-specific BMDs except for lumbar spine BMD.

**Figure 3:**
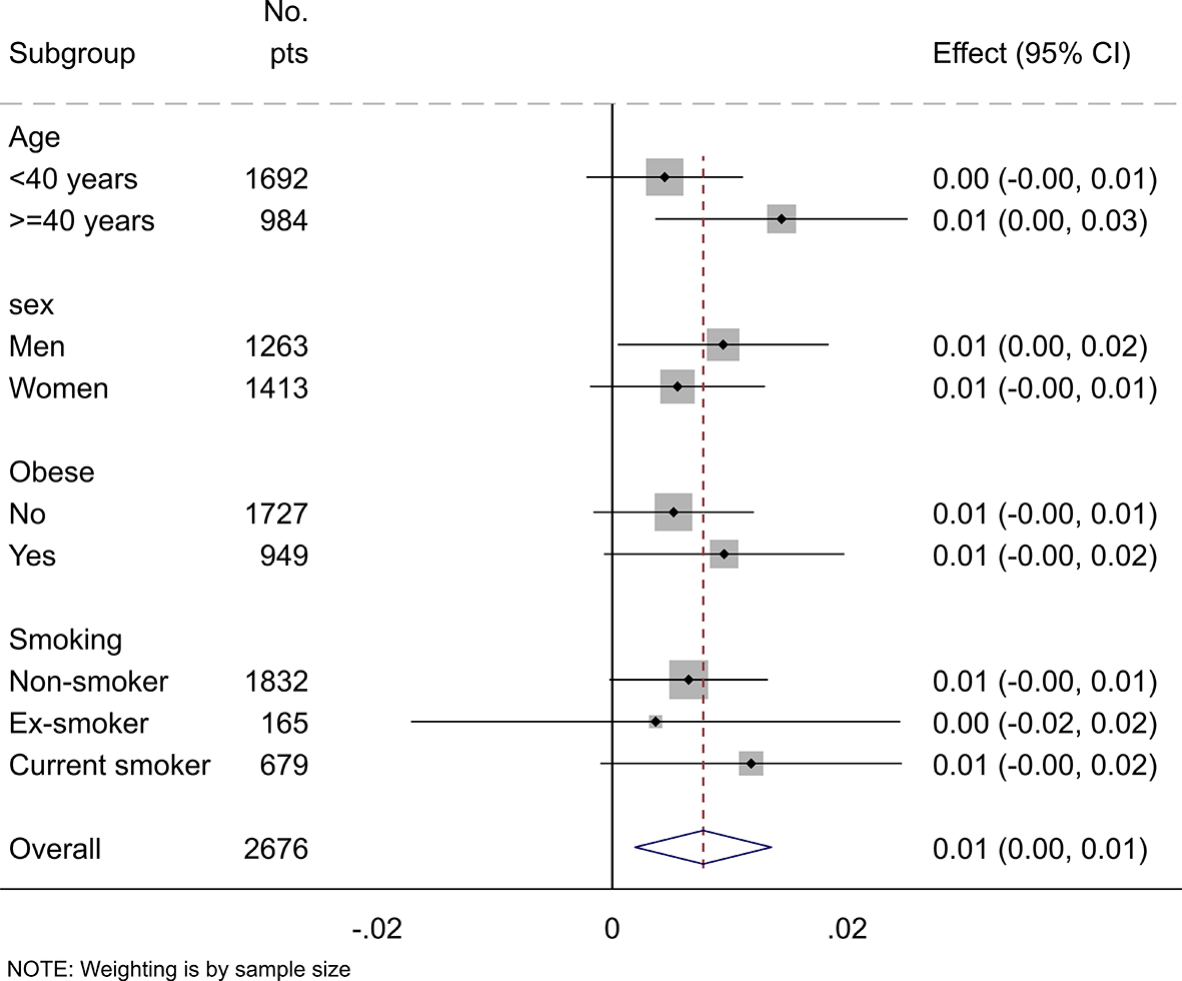
Subgroup analysis of the association between uric acid and total body BMD stratified by age, sex, obesity, and smoking status. Values represent the regression coefficients per SD change of uric acid. Models adjusted for age, gender, BMI, serum vitamin D, alkaline phosphatase, eGFR, and smoking status. Stratification variables were not adjusted in corresponding models.

The subgroup analysis stratified by age, sex, BMI, and smoking status confirmed the overall positive association between SUA, and total body BMD (**Figure 3**). Although the association between SUA and total body BMD was not statistically significant in young adults, women, non-obese, and smokers, no significant interactions between SUA and sex, age, BMI, and smoking were found.

## Discussion

Low BMD is a pathological condition often asymptomatic progressing over years to more serious bone complications like osteoporosis and pathological fractures without any clinical clue (20). Many epidemiological and experimental studies provided inconsistent conclusions about the relation between SUA and BMD. Therefore, this study aimed to investigate the relation between SUA and bone health among healthy Qatari individuals.

The BMD score was used in this study as a measure of bone health by measuring mineral density using dual-energy x-ray absorptiometry (DXA). This technique is considered globally as the standard tool in estimating bone health due to its accuracy and cost-effectiveness. The assessment of other bone components like collagenous and non-collagenous proteins that contribute significantly to bone health is however of limited use (21). Different skeletal anatomical locations were selected in the DXA scan to increase the reliability of the measurements including the total body, hip trochanters, trunk, femoral upper neck, ward’s triangle, pelvis, and lumbar spine BMDs.

The level of SUA was assessed together with BMD and other serological parameters in a relatively large population sample, representative of the healthy Qatari population. A positive association was evident between the quartiles of SUA level and the BMD scores as shown in **Table 1**. As shown in the table, subjects with higher SUA levels had significantly higher BMD at different skeletal sites (p<0.001). The baseline correlation analysis of total body BMD confirmed a significant positive association with SUA level.

Further investigation of multiple variable adjusted associations between quartiles of SUA, total body BMD, and site-specific BMDs confirmed the significant positive association between the quartiles of SUA and all site-specific BMDs in a dose-response pattern after adjustment for age and gender. Age and gender contribute significantly to the BMD as elderly people and post-menopausal women, in particular, who are at higher risks for developing osteoporosis in part, due to increased bone resorption rates and the declining estrogen levels (22).

Further adjustments for age, gender, BMI, serum vitamin D, alkaline phosphatase, and smoking status confirmed high-level of SUA was positively associated with BMD in all skeletal sites except the lumbar spine. Although, the association and the dose-response effect were attenuated after the adjustment indicating the possible confounding factors. This may indicate BMI, age are independent factors significantly contributing to BMD. Similarly in another cross-sectional study, BMI and adiposity were independently associated with BMD as they attenuated the association between SUA and BMD to marginal significance after adjustment (23). Such finding was also confirmed in other studies (24, 25). In the study conducted by Han et al. on a group of Chinese postmenopausal women, SUA was significantly associated with lumber BMD after adjustments for the aforementioned factors in addition to blood pressure, alcohol intake, milk intake, and calcium supplements (26).

The subgroup analysis of the adjusted regression model indicated no significant interaction between SUA and sex, age, BMI, and smoking. The study also confirmed no significant association between SUA and total body BMD in young adults, women, non-obese, and smokers. This finding is supported by several epidemiological studies using different study designs and population samples (23, 24, 26–40) as listed in **Table 2**. These epidemiological studies aimed to elucidate the relation between SUA and bone health. Many cross-sectional studies have confirmed the positive association between SUA and BMD in both genders and all age groups after adjustment for potential confounders (28, 36, 38). In other cross-sectional studies, the positive association was dependant on a significant interaction of SUA level with age and vitamin C level (40), central adiposity, or obesity among postmenopausal women (23, 24, 26). other cross-sectional studies have suggested an additional protective effect of SUA against the development of osteoporosis or osteoporotic fractures (27, 30, 32, 33, 37, 39).

**Table 2:** Studies with a positive association between SUA and BMD in different study designs and populations.

Study	Country	Skeletal site of Bone scan	Sample Size	Participants Type
Ahn et al. (27)	Korea	lumbar spine, femoral neck, total hip, and trochanter	7502	Postmenopausal women
Ishii et al. (28)	Japan	Lumbar spine	615	Peri & Postmenopausal women
Kim et al. (29)	Korea	Osteoporotic fractures	16078	Men aged over 50 years
Lane et al. (30)	USA	Total hip	1680	Men aged over 65 years
Makovey et al. (31)	Australia	Femoral neck, hip, lumber spine, total body	356	Peri & Postmenopausal women
Nabipour et al. (32)	Australia	Lumbar spine and right hip	1705	Men aged over 70 years
Babaei et al. (33)	Iran	Lumber spine and femoral neck	1080	Men and women aged over 70 years
Karimi et al. (36)	Iran	Total body, lumbar spine, and left femoral neck	413	Healthy young adults
Alosami et al. (39)	Iraq	Lumber spine and right femoral neck	151	Postmenopausal women
Han et al. (26)	China	Lumber spine	390	Postmenopausal women
Yao et al. (34)	USA	Lumber spine	4156	Old adults aged over 60 years
Pan et al. (35)	USA	Total body	7320	Healthy young adults
Bonaccorsi et al. (24)	Italy	Hip	358	Pre & Postmenopausal women
Pirro et al. (23)	Italy	Femur	180	Postmenopausal osteoporotic women
Muka et al. (40)	Netherlands	Femural neck and hip bone geometery	5074	Population study (Rotterdam Study)
Chen et al. (37)	China	lumbar spine and left femoral neck	253	Primary osteoporosis patients
Kaushal et al. (38)	India	femur neck, total femur, and lumbar spine	524	Healthy adults

Consistently two longitudinal studies had concluded the positive association between SUA and annual rates of change in BMD at different skeletal sites (31) and lower risk in developing osteoporotic fractures (29). Interestingly, other studies had additionally confirmed the lack of association between SUA and BMD in young females (35) and black Americans (34).

In contrast to these observations, a group of epidemiological studies confirmed no association between SUA and BMD after adjusting confounders in the multiple regression analysis (41). One cross-sectional study in the USA also confirmed no association between SUA and the risk of non-vertebral fracture among gout patients (42). Another cross-sectional study in Italy even concluded higher risks of hip fractures among men with high SUA levels due to the role of inflammatory factors and the involvement of oxidative stress response (43). However, the risk of fracture is not only determined by BMD; besides, physical activity, smoking, nutrition, weight loss, and several hormonal and therapeutic agents might count. In one longitudinal study in Italy despite the baseline relation of high SUA with lower risk with osteoporosis; however, the association was not evident with the follow-up of the participants up to 4 years (44). These conflicting observations may be attributed to the demographic characteristics, confounding factors being controlled, population sample, study designs, and other limitations.

Interestingly, one Mendelian randomization study found no causal effect of SUA or gout with BMD (45). However, randomized Mendelian studies carry many limitations because the level of SUA is not merely determined by genetic factors. Instead, several environmental factors may obviate the genetic influence such as daily intake of purine diet, alcoholism, diuretics, high blood pressure, and high blood sugar (46). Other limitations of randomized Mendelian studies may include multiple biological effects of the genetic variant (pleiotropy) that may independently affect SUA level in addition to the consequences of linkage disequilibrium that involve other confounding genetic variants.

The hypothesized mechanism played by SUA in promoting bone health is attributed to its antioxidant properties. Accumulating evidence suggests the plausible involvement of oxidative stress as a fundamental contributor to age-related bone loss. Oxidative stress inhibits osteoblast cell differentiation and function in mineralizing the bone tissue and may even induce cell death with evident reversal of all changes upon treatment with antioxidants such as Trolox or ascorbic acid (47, 48). Oxidative stress may also stimulate the development and function of osteoclast cells that exhibit bone resorption *via* ROS-mediated mechanisms (49, 50).

Several studies have shown that SUA is a strong endogenous antioxidant that counts nearly for half of the plasma’s antioxidant potential (51). Hence, SUA may promote proliferation and osteogenic differentiation and protect against metabolic bone diseases, such as osteoporosis. This observation was confirmed in human bone mesenchymal stem cells *in vitro*; Interestingly uric acid enhances the proliferation and differentiation of the cells, increases deposition of calcium crystals, and inhibits the adipogenic stem cells of the bone (52). Moreover, uric acid inhibits the formation and function of osteoclast cells *in vitro* with a dose-dependent effect through interference with ROS precursors within the osteoclast cells (27). However, unfavorable effects are elicited with very high levels of uric acid *in vitro* and *in vivo* gout models. At such levels, uric acid is found to inhibit the proliferation and differentiation of osteoblast cells and the toxic effects were reversed upon treatment with anti-hyperuricemia agents like allopurinol (53).

One experimental study confirmed no difference in the BMD and bone biochemical parameters among rat models of hyperuricemia compared to the control rats (41). This observation could be attributed to other confounding antioxidants such as ascorbic acid. Interestingly, unlike in humans, ascorbic acid can be biosynthesized in rodents due to the lack of gluconolactone oxidase enzyme in human beings (54). Like SUA, ascorbic acid is a powerful antioxidant and may confer osteoprotective properties despite low or normal levels of SUA. Thus, SUA can effectively prevent the production of ROS in human osteoblasts and stimulate its differentiation, hence, increasing bone formation and optimize bone health when present at normal levels.

Another finding in this study is the low level of vitamin D among the healthy participants as shown in **Table 1**. Despite the abundant sunshine in the Middle East and Asia compared to Europe and the United States, countries in these areas have reported the highest rate of hypovitaminosis D worldwide. For instance, in Thailand, the prevalence of vitamin D deficiency was found to be in 77% of pre-menopausal women, and it reached up to 90% in India (55, 56). Qatari population has one of the highest prevalence rates of vitamin D insufficiency that is approximately affecting 90.4% of the population (57). Low intake of vitamin D supplements may contribute to the deficiency status among the younger population. Hence, the estimation of vitamin D deficiency/insufficiency becomes mandatory before reporting bone status in young as well as post-menopausal women.

The limitation of this study is attributed to the cross-sectional design where it is difficult to determine a causal relationship between BMD and SUA. Longitudinal studies are necessary to elucidate this relation though it was confirmed in other populations. In addition, many subjects were excluded from the analysis such as patients on corticosteroids treatment and patients receiving chemotherapy for cancer because both conditions may significantly interfere with BMD (57); similarly, multiple pieces of evidence confirmed the relationship between low BMD and chronic disorders involving the vital organs (heart, lung, liver, kidney, brain), diabetes, high cholesterol level, high blood pressure, kidney diseases, stroke, arthritis, osteoporosis, fractures, Parkinson disease, thyroid disease, hysterectomy, Hodgkin lymphoma, breast, prostate, and lung cancers. Therefore, the findings in this study may not apply to individuals with above conditions. The study mandates more experimental studies *in vitro* and *in vivo* controlling confounding factors such as ascorbic acid and other antioxidants to unravel the underpinning mechanisms and pave the way for future therapeutic approaches.

## Conclusion

The study concluded the possible osteoprotective properties of SUA among healthy Qatari adults. The observed association was not confirmed in non obese, females, young adults and smokers. The healthy Qatari population had also significantly low levels of vitamin D. further efforts are required to investigate the plausible molecular mechanisms of the effects involving uric acid in bone metabolism and to correct the evident state of vitamin D deficiency in Qatar.

## Data Availability Statement

Restrictions apply to the availability of these data. Data was obtained from Qatar Biobank (https://www.qatarbiobank.org.qa/) and are available from Qatar Biobank upon request.

## Ethics Statement

The study involved human participants and was reviewed and approved by Institutional review board (IRB) at Qatar university (E -2018-QBB-RES-ACC-0112-0054). The patients/participants provided their written informed consent to participate in this study.

## Author Contributions

WI conceptualized the topic, wrote and edited the manuscript, contributed in data curation and the statistical analysis. NY edited the reviewed the manuscript and collected the data. ZS completed the statistical analysis, reviewed the manuscript. MA-M project administration, supervision, and reviewing the manuscript. All authors contributed to the article and approved the submitted version.

## Funding

This study was funded by Qatar University, grant # QUST-1-CHS-2019-16.

## Conflict of Interest

The authors declare that the research was conducted in the absence of any commercial or financial relationships that could be construed as a potential conflict of interest.
